# Coffee Chlorogenic Acids Incorporation for Bioactivity Enhancement of Foods: A Review

**DOI:** 10.3390/molecules27113400

**Published:** 2022-05-25

**Authors:** Alexis Rojas-González, Claudia Yuritzi Figueroa-Hernández, Oscar González-Rios, Mirna Leonor Suárez-Quiroz, Rosa María González-Amaro, Zorba Josué Hernández-Estrada, Patricia Rayas-Duarte

**Affiliations:** 1 Tecnológico Nacional de México/Instituto Tecnológico de Veracruz, M.A. de Quevedo 2779, Col. Formando Hogar, Veracruz 91897, Mexico; alexis.rojas@okstate.edu (A.R.-G.); oscar.gr@veracruz.tecnm.mx (O.G.-R.); mirna.sq@veracruz.tecnm.mx (M.L.S.-Q.); zorba.he@veracruz.tecnm.mx (Z.J.H.-E.); 2 Robert M. Kerr Food & Agricultural Products Center, Oklahoma State University, 123 FAPC, Stillwater, OK 74078, USA; 3 CONACYT-Tecnológico Nacional de México/Instituto Tecnológico de Veracruz, Unidad de Investigación y Desarrollo en Alimentos, M. A. de Quevedo 2779, Veracruz 91897, Mexico; claudia.fh@veracruz.tecnm.mx; 4 CONACYT-Instituto de Ecología, A.C., Carretera Antigua a Coatepec 351, Col. El Haya, Xalapa, Veracruz 91073, Mexico; rosa.gonzalez@inecol.mx

**Keywords:** chlorogenic acids, coffee, 5CQA, functional foods, biological activity

## Abstract

The demand of foods with high antioxidant capacity have increased and research on these foods continues to grow. This review is focused on chlorogenic acids (CGAs) from green coffee, which is the most abundant source. The main CGA in coffee is 5-O-caffeoylquinic acid (5-CQA). Coffee extracts are currently the most widely used source to enhance the antioxidant activity of foods. Due to the solubility of CGAs, their extraction is mainly performed with organic solvents. CGAs have been associated with health benefits, such as antioxidant, antiviral, antibacterial, anticancer, and anti-inflammatory activity, and others that reduce the risk of cardiovascular diseases, type 2 diabetes, and Alzheimer’s disease. However, the biological activities depend on the stability of CGAs, which are sensitive to pH, temperature, and light. The anti-inflammatory activity of 5-CQA is attributed to reducing the proinflammatory activity of cytokines. 5-CQA can negatively affect colon microbiota. An increase in anthocyanins and antioxidant activity was observed when CGAs extracts were added to different food matrices such as dairy products, coffee drinks, chocolate, and bakery products. The fortification of foods with coffee CGAs has the potential to improve the functionality of foods.

## 1. Introduction

**Figure 1:**
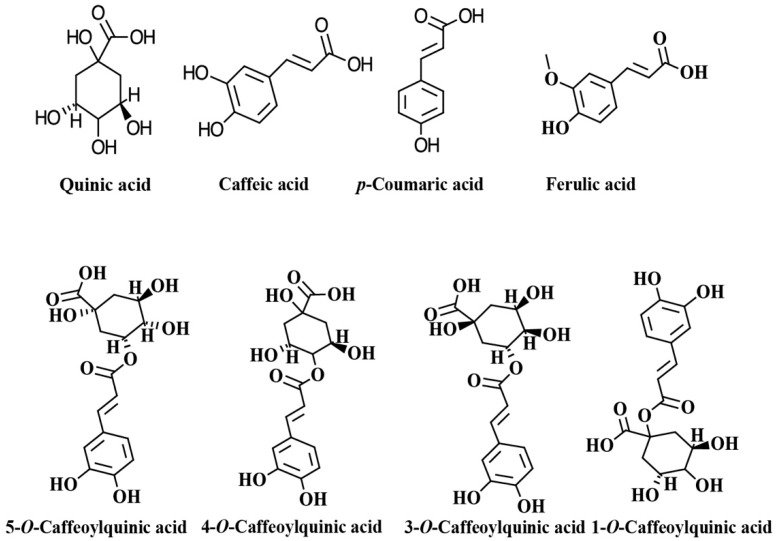
Chemical structures of main chlorogenic acids (CGAs) and isomers of caffeoylquinic acid.

The chlorogenic acids (CGAs) are a class of phenolic compounds widely distributed in various plants sources such as fruits, vegetables, coffee beans, tea, apples, and wine [1,2,3]. CGAs are esters of quinic acid (QA) and one trans-cinnamic acid residue such as caffeic acid (CA), *p*-coumaric acid (*p*-CoA), and ferulic acid (FA), which are known as caffeoylquinic acids (CQAs), *p*-coumaroylquinic acids (*p*-CoQAs) and feruloylquinic acid (FQAs) [1,2,3,4,5]. Caffeoylquinic acid may theoretically form four isomers, but only three are present in plants: 3-*O*-caffeoylquinic acid (3-CQA), neochlorogenic acid (5-*O*-caffeoylquinic acid, 5-CQA), or cryptochlorogenic acid (4-*O*-caffeoylquinic acid, 4-CQA). The most common isomer, 5-CQA, is an ester composed of caffeic acid and (−)-quinic acid and referred as chlorogenic acid [4,6]. According to the number of caffeoyl groups attached to the quinic acid, these CQAs can be classified into monophosphoylquinic acids (MCQAs), dicaffeoylquinic acids (DCQAs), tricaffeoylquinic acids (TCQAs), and tetracaffeoylquinic acids (tetra-CQAs) [3]. The chemical structures of the main CGAs are shown in Figure 1. The structural diversity and broad bioactivities of CGAs have increasingly attracted the attention of researchers [4,5,7,8,9,10]. It has been demonstrated that these compounds are potent antioxidants and may also exert other physiological activities. For example, there is evidence that CGAs possess a wide variety of bioactivities, such as antiparasitic [11], antibacterial [12], anti-inflammatory [13], neuroprotective [14], anticancer [15], antiglycemic [16], and antiviral [17]. In addition, it has been demonstrated that CGAs have therapeutic effects in the prevention and treatment of some chronic and cardiovascular diseases [5,18,19]. This review aims to describe the main biological activities attributed to coffee CGAs, and their bioavailability and potential addition to different food matrices to obtain functional foods. 

## 2. Dietary Sources of Chlorogenic Acids (CGAs)

CGAs are a large family of esters of quinic acid and trans-cinnamic acids; up to date, at least 71 different chemical compounds are identified from different plants sources such as fruits, vegetables, coffee beans, tea, apples, artichoke, eggplant, and grapes [4,20,21]. However, those found in the highest concentration in plants are caffeoylquinic acids (CQAs), specifically mono- and di-CQAs, as well as the different isomeric forms of feruloylquinic acids (FQAs) [21,22]. Meinhart et al. [23] analyzed the CGAs concentration (CA, 3-CQA, 4-CQA, 5-CQA, 3,4-DQA, 3,5-DQA and 4,5-DQA) of 100 plants commonly used in Brazil as infusions. In their study, the highest concentrations of CGAS were yerba mate (*Ilex paraguariensis*), white and green tea (*Camellia sinensis*), and winter’s bark (*Drimys winteri*). A study of the presence of CGAs in 53 vegetables consumed in Southern Brazil reported the highest concentrations of 3-CQA, 5-CQA, and 4-CQA in collard greens and chicory whereas the highest concentration of 3,4-DQA, 3,5-DQA, and 4,5-DQA were found in bay leaves and mustard [20]. At present, green coffee beans and yerba mate are recognized as the most important plant sources of CGAs, accounting for up to 6 to 12% in the case of green coffee and 9% for mate. 5-CQA is the most abundant CGA in green coffee beans, with a concentration of about 100 mg/g (dry basis), representing 76 to 84% of the total content of CGAs [4]. The main food sources of CGAs are shown in Table 1. 

### Coffee as a Source of CGAs

Coffee is one of the most widely consumed beverages in the world. This infusion contains several compounds that can exert beneficial biological activities for human health. Many beneficial effects have been investigated, mainly attributed to caffeine and other substances, such as polyphenols, mainly chlorogenic acids [1,34]. There are at least 30 different types of CGAs found in coffee, and this includes caffeoylquinic acids (CQAs), dicaffeoylquinic acids (DCQAs), tricaffeoylquinic acids (TCQAs), feruloylquinic acids (FQAs), and *p*-coumaroylquinic acids (*p*-CoQAs) [35]. One cup (200 mL) of coffee brew contains between 20–675 mg of CGAs depending on the variety of coffee and brewing method [36].

Whether green or roasted, the beneficial health effects of coffee have been attributed to the high content of CGAs and the antioxidant activity provided by the phenolic compounds in green coffee in addition to the those produced during the roasting process [37]. The concentration of active polyphenols inside green coffee depends on the variety of the bean and its geographical origin; in beverages, it also depends on the brewing process [38]. During the coffee roasting process, phenolic compounds undergo a series of intermolecular and intramolecular reactions and interactions [21]. Higher concentration of CGAs in lightly roasted coffee over dark roasted coffee has been established; however, the highest concentration of CGAs is found in green coffee beans [39]. In green coffee, CQAs alone account for up to 80% of the total CGAs and among CQAs, 5-CQA account for almost 60%. Thus, 5-CQA is the most studied isomer of the CGAs and is responsible for the bitter and astringent taste in coffee [25].

## 3. Extraction of Chlorogenic Acids (CGAs) from Coffee

The extraction recovery of a wide variety of compounds from vegetal species is a critical step in the production of bioactive substances. The chemical properties of CGAs such as thermal stability, solubility, and oxidation-reduction reactions, need to be considered when combined with other substances [21]. Studies focused on the development of new extraction methods of CGA’s have been made over the last couple of decades, mainly focusing on increasing mass transfer and extraction yields while minimizing the use of toxic organic solvents and energy consumption [40,41,42,43,44,45,46].

### 3.1. Organic Solvent Extraction

Madhava-Naidu et al. [46] extracted green coffee CGAs by sterilizing the beans at 120 °C for 20 min and Soxhlet extraction with hexane at different rates. The samples were then separated in glass columns and extracted with selected solvents at different ratios (80:20, 70:30, and 60:40) using mixtures of isopropanol and water. They obtained the best extraction yield (29.1%) and a CGAs content of 29.7% when they used a 60:40 isopropanol-water ratio in Robusta coffee, whereas in Arabica coffee the yield of extraction and CGAs content were 27.3 and 30.2%, respectively. Suárez-Quiroz et al. [40] compared four different methods of CGAs extraction using different solvents (water, aqueous methanol, aqueous isopropanol, and ethyl acetate). The extract yield values were not significantly different, demonstrating the high solubility of CGAs in organic solvents. CGA’s extraction from green coffee (*C. arabica*) using water at 80 °C and activated carbon were not significantly different from the values of the previous investigation [47]. Thus, activated carbon is a suitable and more eco-friendly extraction method with a minimum of 97% CGAs purity in the extract was reported in this study [47]. 

Dibert et al. [48] tested the effect of different physicochemical parameters (temperature, particle diameter, and solvent-mass ratio) when extracting CGAs from green coffee beans with a methanol-water extraction (70:30 ratio) at three different temperatures (30, 40, and 50 °C). The highest yield of extraction of CGAs (18.1%) was obtained at 40 and 50 °C, when a mass-solvent ratio of 1:4 *w*/*v* was used. By increasing the mass ratio of green coffee beans an improvement in the yield of extraction of CGAs can be achieved. 

### 3.2. Pulsed Electric Field Extraction 

Bilge et al. [45] evaluated the effect of pulsed electric field on green and roasted C. arabica beans as a pretreatment by exposing them to monopolar pulses of 2 Hz with an interval of 0.5 s and generating an electric field of 28 kV/10 cm with water at 20 °C. The use of an electric field increased radical scavenging activity up to 31% and 11%, for green and roasted coffee beans, respectively, compared to untreated samples confirming that using electric pulses as a pretreatment before extraction can enhance the phenolic content extraction and reduce Maillard reaction products that occur at high temperatures of extraction and during the coffee roasting process. Phongsupa et al. [44] studied the extraction of CGAs by pulse electric field induction over *C. arabica*. The number of pulses and concentrations for this study was set to 1000 pulses at 5 kV and 62.7% methanol-water solution as solvent. The mass-solvent ratio with the most effective extraction was 0.75 g/mL and 30 s of blending which had the CGAs content of 9.8 μg/g. However, the results obtained in this study showed that an increase in the sample-solvent ratio leads to a higher concentration of CGAs [45].

## 4. Biological Activities of CGAs 

**Figure 2:**
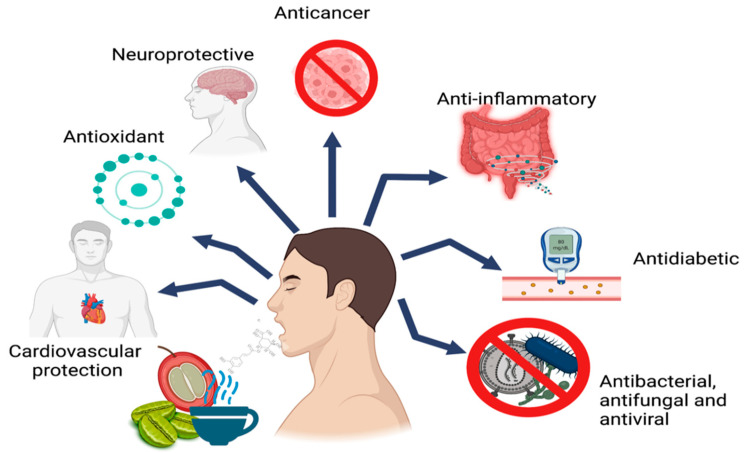
Main biological activities attributed to CGAs.

Several studies have associated CGAs with beneficial health properties, such as antioxidant, antiviral, antibacterial, anticancer, and anti-inflammatory activity [4,5,6,49]. It has also been shown that it can modulate the gene expression of antioxidant enzymes and reduce the risk of cardiovascular disease by suppressing the expression of P-selectin in platelets [49]. In addition, CGAs can reduce the relative risk of type 2 diabetes and Alzheimer’s disease [6,50,51,52,53,54]. The main biological activities attributed to CGAs are shown in Figure 2.

Some of these properties are well recognized and demonstrated by in vitro and in vivo studies, such as antioxidant activity. However, other bioactivities of interest in recent years, although not yet well demonstrated, such as the potential anti-obesity [52,55,56,57,58,59,60] or prebiotic [61,62,63] properties of CGAs. In addition, it has also been shown that CGAs can modulate the activity of glucose-6-phosphatase, an enzyme involved in glucose metabolism, and therefore it may have a positive effect on diabetes management [64].

Furthermore, it is important to highlight that these biological activities are dependent on the CGA’s stability. CGAs are particularly susceptible to environmental conditions, such as solvent type, pH, temperature, and light. These factors must be considered during the CGAs extraction. Moreover, the concentration of these compounds in plants is low. For this reason, the methodologies used for the CGAs extraction from plant sources must be efficient to guarantee the necessary concentration of CGAs to exert their biological activity. 

### 4.1. Antioxidant Activity

There is a strong correlation between oxidative stress and the development of various degenerative diseases such as cancer and other aging-related diseases [65,66]. Extensive in vitro and in vivo studies have been performed to evaluate the antioxidant activity of CGAs [67]. As a result, CGAs are known to exhibit a radical scavenging effect similar to ascorbic acid [68]. In addition, CGAs can chelate transition metals such as Fe^2+^ to scavenge free radicals and disrupt chain reactions [21]. Studies have shown that CGAs may prevent the oxidation of low-density lipoproteins (LDL) induced by different oxidizing agents [69,70], as well as prevent DNA damage in vitro [71]. 5-CQA, which is the most important CGA in coffee, can scavenge 1,1-diphenyl-2-picrylhydrazyl radicals (DPPH), superoxide anions (O_2_^•−^), hydroxyl radicals (^•^ OH), and peroxynitrite (ONOO^−^) [72,73,74], and protect DNA from damage caused by oxidative stress in different studies [67,75].

Therefore, there is enough evidence to support that CGA_S_ can inhibit the formation of reactive oxygen species and play a beneficial role in preventing oxidative and aging-related diseases [65,66]. However, studies indicate that these compounds may also act as potent pro-oxidants. Therefore, depending on their concentration, the presence of free transition metal ions, or their redox state, the antioxidant and pro-oxidant properties of CGAs can be modified [76,77,78]. 

### 4.2. Anti-Inflammatory Activity

Inflammation is a complex physiological process of tissue injury caused by exogenous or endogenous sources [67]. A prolonged unregulated inflammatory process can induce tissue damage and is the cause of many chronic pathologies, such as diabetes, alcoholic liver, chronic kidney disease, and cardiovascular and neurodegenerative diseases [79,80]. CGAs, mainly 5-CQA, have been shown anti-inflammatory activity by reducing pro-inflammatory cytokines, due to modulation of key transcription factors, such as tumor necrosis factor-alpha (TNF-α) and interleukins, such as IL-8 [67,81]. Another study performed in murine RAW264.7 macrophages showed that 5-CQA decreased lipopolysaccharide (LPS)-induced cyclooxygenase (COX-2) up-regulation at both the protein and mRNA levels, suggesting that 5-CQA might exert anti-inflammatory effects through inhibition of prostaglandin E2 (PGE2) production [82]. It has also been reported that CFA can enhance the wound healing process [67]. In a study with diabetic rats, oral administration of 5-CQA increased hydroxyproline concentrations and decreased malondialdehyde/nitric oxide levels in wound tissues. In addition, it allowed elevation of reduced glutathione [83,84]. Topical administration of 5-CQA-containing hydrogels to mouse skin wounds significantly reduced the size of the wound area in the inflammatory phase, improving the healing process [85]. 

### 4.3. Neuroprotective Activity

Alzheimer’s disease is a neurodegenerative disease characterized by progressive deterioration of learning, memory, and other cognitive deficits, along with the extracellular deposition of β-amyloid peptides into the brain leading to neuroinflammation, synaptic loss and neuronal death [86,87]. According to Alzheimer Association [88], in 2050, the number of people aged 65 and older with Alzheimer’s disease will reach 12.7 million. Several studies found an inverse relationship between coffee consumption and the development of Alzheimer’s disease, suggesting its possible use in managing treatments [86,89,90,91,92]. The neuroprotective mechanisms of coffee are suggested to be related to the anti-inflammatory effects of caffeine and CGAs on A_1_ and A_2_ receptors. In addition, it reduces toxic deposits of β-amyloid peptides in the brain, which is a distinctive feature in Alzheimer’s patients [6,90,92,93]. Furthermore, some coffee compounds could inhibit brain acetylcholinesterase and butyrylcholinesterase (causing a delay in the degradation of acetylcholine and butyrylcholine), resulting in the prevention of oxidative stress-induced neurodegeneration due to their high antioxidant activity [6,90,94].

On the other hand, murine model trials have shown a significant association between the consumption of CGAs and the prevention of the development of degenerative diseases and aging [6,95,96,97,98]. The effect of phenolic compounds from coffee on human cognitive function has not been well studied [99]. However, the number of in vitro studies concerning the neuroprotective effects of polyphenols is rapidly increasing. It has been demonstrated that intraperitoneal injections of 5-CQA reduced oxidative damage in the cerebellum of rats exposed to methotrexate, a drug with serious side effects used to treat some types of cancer, rheumatoid arthritis, and psoriasis [100]. In the same study, these researchers also observed that application of 5-CQA decreased lipopolysaccharide (LPS)-induced IL-1β and (TNF-α) release in the substantia nigra, indicating neuroprotective effects of 5-CQA on neurodegenerative diseases caused by proinflammatory cytokines [100]. Taram et al. [101] studied the neuroprotective effects of 5-CQA and caffeic and ferulic acids on rat cerebellar granule neuron cultures. This research proposed that caffeic acid showed enhanced neuroprotection against a wide range of stressors compared to the other compounds evaluated. Thus, the authors suggest that caffeic acid could be a promising candidate in preclinical models of neurodegeneration [101].

### 4.4. Anticancer Activity

The antimutagenic properties of CGAs was demonstrated decades ago [102]. This activity is partially related to the antioxidant activity of these compounds since the overproduction of oxygen free radicals leads to oxidative DNA damage. This damage is leading cause of the proliferation of several types of cancer, such as breast, colon, bladder, pancreatic, liver, skin, and prostate cancer [103]. Dietary polyphenols, including CGAs, can protect the initiation of tumor processes by inhibiting DNA lesions caused by both free radicals and carcinogens [104]. Indeed, some epidemiological studies demonstrated an inverse relationship between coffee consumption and the risk of certain types of cancer. This effect has been associated with the intake of CGAs [105,106,107]. Several mechanisms have suggested that CGAs may have a chemopreventive effect [80]. Among those, modulation of the expression of enzymes involved as endogenous antioxidant defenses, in DNA replication, as well as in cell differentiation and aging are prominent [104,108]. Moreover, metal chelation, inactivation of reactive compounds, and changes in metabolic pathways have been proposed to impact anticancer activity significantly [109]. Boettler et al. [110] demonstrated by in vitro and in vivo assays that coffee-derived CGAs can induce a cellular and tissue protection mechanism against carcinogenesis via the Nrf2/ARE pathway. This pathway regulates the expression of S-transferases (GST), γ-glutamate-cysteine ligase (γGCL), NAD(P)H: quinone oxidoreductase 1 (NQO1), and heme oxygenase (H01). In another study by Feng et al. [108] using mouse epithelial JB6 cells, it was found that 5CQA had a protective effect against carcinogens. This effect was due to its ability to decrease the generation of free radicals and stimulate glutathione-S-transferase activity.

### 4.5. Antidiabetic Activity

According to International Diabetes Federation [111] diabetes (type 1 and 2) is one of the fastest-growing global health emergencies of the 21st century. It was estimated that 537 million adults aged 20–79 years are currently living with diabetes and type 2 diabetes mellitus (T2DM) is the most common type of diabetes, accounting for over 90% of all diabetes worldwide [111]. Several studies have demonstrated an association between moderate consumption of coffee and a lower risk of developing T2DM. This was observed in all sexes, obesity levels, and geographic locations [112,113,114,115,116,117,118,119]. This effect has been attributed to the bioactive compound 5-CQA. Through a meta-analysis, Huxley et al. [120] concluded that daily consumption of three to four cups of coffee decreased the risk of T2DM by 25%.

**Figure 3:**
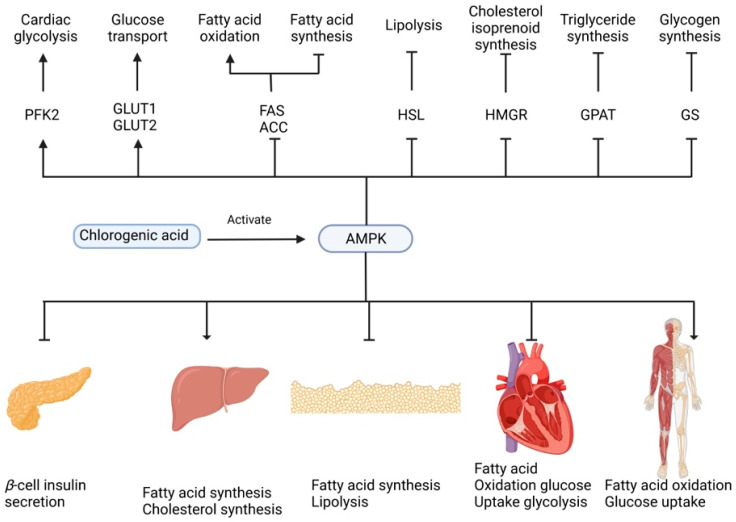
5CQA-mediated regulation of glucose and lipid metabolism through activation of the AMPK pathway.

Furthermore, Bakuradze et al. [121] suggested that consumption of three to four cups of coffee per day could reduce oxidative damage, body fat mass, and energy/nutrient intake and that these effects were partially attributed to CGAs. Shearer et al. [122] studied the effects of regular and decaffeinated coffee (with CGAs) consumption for 28 days on insulin functions, in vivo using a rat model. They observed that the ingestion of decaffeinated coffee improves insulin-stimulated disposal in the high-fat-fed and insulin-resistant rats. Other suggested mechanisms of CGAs are related to the improvement of glucose and lipid metabolism by activating of AMP activated protein kinase (AMPK) [119], as shown in Figure 3. AMPK is a master sensor and regulator of cellular energy balance. This enzyme is activated by diverse pathological, metabolic, and pharmacological stressors such as hypoxia, exercise, thiazolidinediones, and metformin. This activation provokes the translocation of glucose transporter type 4 (GLUT4) from intracellular membranes to plasma and, therefore, the increase of glucose transport [119,123,124].

### 4.6. Cardiovascular Protection Activity 

Currently, cardiovascular diseases (CVDs) comprise one of the leading causes of death and disability worldwide. The incidence of various chronic CVDs, including stroke, atherosclerosis, hypertension, ischemic heart disease, and heart failure, probably continues to increase [4]. Some risk factors, such as smoking, high blood pressure, hyperlipidemia, and hyperglycemia, have been reported to contribute, partially, to the development of CVDs [4]. According to the World Health Organization (WHO), ischemic heart disease is the leading cause of death worldwide, accounting for 16% of deaths worldwide (8.9 million people) [125]. Recently, many studies have shown that the consumption of CGAs-rich foods may be recommended to prevent CVDs [49,119,126,127,128]. The high antioxidant and anti-inflammatory activity of CGAs can improve endothelial dysfunction and reduce insulin resistance which could be critical mechanisms to enhance the cardiovascular protection attributed to these compounds, as shown in a large number of in vitro and in vivo studies [67]. Taguchi et al. [129] observed that CGAs could improve endothelial function through by releasing of vasoactive molecules such as nitric oxide. This effect was studied in streptozotocin-treated diabetic rats. On the other hand, CGAs could decrease blood pressure by the following proposed mechanisms: (i) stimulation of nitric oxide production through the endothelium-dependent pathway [130], (ii) reduction of free radicals through decreased expression and activity of NADPH oxidase [131], and (iii) through inhibition of the angiotensin-converting enzyme (ACE) [67]. 

### 4.7. Antibacterial, Antifungal, and Antiviral Activity 

The antimicrobial (bacteriostatic and bactericidal) effects of 5-CQA and coffee extracts on various types of detrimental microorganisms that may grow in different parts of the body, from oral bacteria causative of caries to harmful intestinal bacteria, are well known. Roasted *C. arabica* and *C. canephora* extracts and brews exhibited antibacterial activity against *Streptococcus mutans* and other oral types of bacteria [132,133]. Furthermore, 5-CQA can have a positive affect against the adverse microbiota present in the colon. Therefore, this chlorogenic acid can be used as a preservative and food additive [10]. For this reason, CGAs, mainly 5-CQA, could be potential natural antibacterial, antifungal and antiviral agents [2]. For example, 5-CQA exhibited a broad-spectrum antimicrobial activity against Gram-positive (*Streptococcus pneumoniae*, *Staphylococcus aureus*, and *Bacillus subtilis*) and Gram-negative (*Escherichia coli*, *Shigella dysenteriae*, and *Salmonella typhimurium*) pathogenic bacteria by increasing the membrane permeability, leading to plasma membrane barrier dysfunction, as well as leakage of nucleotide [134,135]. The suggested mechanism by which 5-CQA provokes the membrane disruption could involve the perturbation of the membrane lipid bilayer, resulting in cell leakage and dissipation of the membrane electrical potential [4,135]. 

In addition, Sung and Lee [136] studied the antifungal properties of 5-CQA against *Candida albicans*, a pathogenic yeast. They suggested that this compound could exert antifungal activity by disrupting the cell membrane structure and consequently, it can be used as an option for fungal treatment. In several studies, both caffeic acid and 5-CQA have demonstrated multiviral activities against herpes simplex virus (HSV) types 1 and 2 [137], adenovirus [138], and HIV [139]. 

### 4.8. Other Bioactivities

#### 4.8.1. Hepatoprotective Activity 

The beneficial effects of coffee on liver diseases, in general, have been reported in several studies [140,141,142] for example, cirrhosis and hepatitis B and C [142]. Hepatic injury may be due to multiple factors, such as viral hepatitis, obesity, excessive alcohol consumption, and iron overload [67]. On the other hand, according to a meta-analysis of 16 human studies, coffee consumption (2 cups per day) decreased the risk of developing liver cancer by 40% compared to no coffee consumption [143,144]. The suggested mechanisms of hepatic protection were the prevention of cell apoptosis and oxidative stress damage due to the activation of natural antioxidant and anti-inflammatory systems [145,146]. These protective mechanisms have been mainly related to CGA [147] and caffeine [148], among other components of coffee.

#### 4.8.2. Potential Prebiotic Activity

According to the International Scientific Association for Probiotics and Prebiotics (ISAPP), a prebiotic definition is “a substrate that is selectively utilized by host microorganisms conferring a health benefit” [149]. Usually, well-established prebiotics are carbohydrate-based, but other substances such as polyphenols and polyunsaturated fatty acids transformed into their respective conjugated fatty acids can potentially fit into this new prebiotic definition, provided there is sufficient evidence of their positive effect on the host [149]. The consumption of prebiotic foods or compounds selectively favors the growth of probiotic and other health-promoting microorganisms in the gut, especially *Bifidobacterium* and *Lactobacillus* [150,151,152]. Thus, indirectly, the health benefits of prebiotics are the following: (i) production of short-chain fatty acids that lower luminal pH, (ii) stimulation of the growth of beneficial intestinal bacteria and suppression of pathogenic bacteria [151,152], (iii) stimulation of the immune system [153,154], (iv) prevention of colon cancer [155], (v) decrease the prevalence to develop diabetes [156,157], and (vi) increased calcium absorption [158]. Furthermore, Kellow et al. [159] observed that dietary supplementation with prebiotics could reduce or delay the accumulation of advanced glycation end products (AGEs) formed through the Maillard reaction in individuals at high risk for type 2 diabetes and improve or restore the microbial balance within the gastrointestinal tract, potentially reducing AGE absorption.

Several studies have suggested that the non-absorbed part of 5-CQA and caffeic acid in the human gastrointestinal tract serves as a substrate for beneficial intestinal microbiota, thus stimulating their growth [160,161]. Whereas the bifidogenic effect of 5-CQA would seem consensus [61,62], the effect of 5-CQA on *Lactobacillus* growth remains debatable, as only selected strains can utilize it as a substrate [62,63]. Furthermore, Parkar et al. [61] reported an increase in short-chain fatty acids (butyric, acetic, and propionic acid) promoted by 5-CQA. Nevertheless, it has also been observed that 5-CQA promotes the growth of *Firmicutes* and *Bacteroides*, and *Clostridium*. Moreover, an inhibitory effect on the growth of *E. coli* has only been demonstrated in one study [135]. Therefore, more studies are needed to validate the effect of 5-CQA as a prebiotic.

## 5. Bioavailability of CGAs

Numerous studies have shown the potential health benefits of CGAs. Consequently, evidence of the absorption and bioavailability of CGAs is needed to evaluate these compounds’ health benefits fully. However, the absorption and bioavailability of CGAs are controversial due to the significant interindividual differences regarding their utilization, metabolism, and excretion found in scientific and clinical studies.

### 5.1. Absorption of CGAs

Past studies have considered that 5-CQA, such as other phenolic compounds, could be poorly absorbed by the digestive system [162]. However, other studies have shown that a part of this compound can be absorbed intact in the stomach and/or small intestine [163,164]. It is now known that, on average, almost one third of the 5-CQA obtained from the diet is absorbed from the gastrointestinal tract into the bloodstream, although its absorption varies among humans [162,165,166,167,168]. For example, after coffee consumption, two plasma concentration peaks of CGAs corresponding to 5-CQA and DCQA were found at 0.5 to 1.0 and 1.5 to 4.0 h, respectively [163]. Furthermore, Mubarak et al. [169] reported a higher concentration of intact 5-CQA in plasma at 2.5 h in all healthy volunteers following intake of pure 5-CQA (400 mg, approximately corresponding to two cups of coffee). Therefore, it has been suggested that 5-CQA is absorbed through at least two pathways. One pathway may involve immediate absorption of intact 5-CQA in the stomach and/or upper gastrointestinal tract, whereas the other involves slow absorption of intact 5-CQA throughout the small intestine [4]. Additionally, Erk et al. [170] reported that a high intake of 5-CQA from coffee could modify gastrointestinal transport and influence its absorption and metabolism.

### 5.2. Metabolization of CGAs

The human metabolism of CGAs is somewhat complex but well defined. The main pathways of CGAs metabolism are as follows: (i) absorbed non-transformed, (ii) absorbed in the stomach or small intestine, with or without hydrolysis, and then conjugated (sulfate, glucuronide, or methyl) or otherwise metabolized (hydrogenated, α- or β-oxidized, conjugated with glycine), (iii) undergo gut microbiota-mediated metabolism, after which the microbial catabolites are absorbed without further change, or (iv) undergo metabolism via the intestinal microbiota, after which the microbial catabolites are absorbed and undergo mammalian phase II metabolism (conjugation with glucuronide, sulfate, methyl, or glycine) or are otherwise metabolized (hydrogenated, dehydrogenated, α- or β-oxidations) [171]. Thus, it has been found that 33% of the total intake of 5-CQA from the diet is absorbed intact, unhydrolyzed, in the stomach or upper intestine and subsequently passes into the bloodstream. About 7% of the total intake of 5-CQA is absorbed through the small intestine by hydrolysis to CA and QA. Furthermore, part of the metabolism of 5-CQA is mediated by the colonic microbiota. In some studies, traces of 5-CQA have been found in the urine (0.3–2.3%) after ingesting foods with a high content of this phenolic compound, indicating that the absorption of intact 5-CQA is intensively metabolized [163,165]. It is important to highlight that the absorbed part of 5-CQA and its metabolites can induce various physiological effects through the bloodstream, while unabsorbed 5-CQA can induce biological effects throughout the digestive tract, such as modification of the gut microbiota [62,165].

## 6. Incorporation of CGAs into Food Matrices

The growing demand for healthier foods and better lifestyles is relevant for consumers nowadays. Nutrition scientists and food scientists have established that the best way to enrich and fortify food products in overall nutrient intake with minimum side effects is by using extracts and compounds from natural sources (cereals, greens, fruits, etc.) [172]. Chlorogenic acids obtained from distinct vegetal species and their wastes can be used as natural ingredients for different food products as shown in Table 2. Plant foods such as vegetables and fruits are the main source of calories, carbohydrates, and other essential compounds for the human body and play an important role in human health, such as polyphenols [173]. To increase the intake of polyphenols and the level of acceptance among consumers around the globe, studies on food technology using polyphenols have been increasing over the last couple of decades. Polyphenols are a very significant source of phytochemicals which have been used by the pharmaceutical industry for many years in a wide variety of products [174].

Corso et al. [176] studied the antioxidant properties of CGAs and enriching coffee itself. CGAs extract was obtained by a series of percolation stages with pressure water at 180 °C in the first and 100 °C at the final stages. The extract was freeze-dried and added to instant coffee formulations. It was added to obtain a concentration of 7% polyphenols in four different instant coffee formulations. The green coffee extract had 14% CQAs, and 5-CQA was the most abundant [25]. The formulation of instant coffee increased its 5-CQA content and showed 3.18 g/100 g compared to the control 1.20 g/100 g. Moreover, CGAs addition increases antioxidant activity in the instant coffee (evaluated by the ABTS and Folin methods); for enriched coffee, the antioxidant activity was in a range of 30.9–32.0 g of Trolox/100 g, whereas for the control, the content was 24.0–25.6 g of Trolox/100 g. Additionally, the authors reported that the antioxidant activity is not significantly affected by the roasting process. Since the polyphenols reduction (CGAs) is balanced by increasing melanoidin content, Vignoli et al. [182] had similar results. Moreover, no significant difference was found when the sensory evaluation was performed, and all formulations were accepted, obtaining average scores from 6.6 to 7.7 on a hedonic 10-point scale.

Bakery products are well-known sources of energy and nutrients such as carbohydrates, proteins, minerals, and vitamins. However, these also lack antioxidant-rich polyphenolic compounds, fiber, minerals, vitamin B6, thiamine, folate, vitamin E, and some phytochemicals, mostly because the bakery products are formulated with refined wheat flour [183,184]. A study to determine the functional and technological properties of GCE rich in hydroxycinnamic acids (CGAs) in wheat flour and bread was made by Mukkundur et al. [178]. Three levels of GCE obtained from defatted and decaffeinated *C. canephora* green coffee beans were added at 1, 1.5, and 2% on wheat flour. A decrease in total polyphenols (TTP), CGAs, and radical scavenging activity (RSA), 20.0, 36.2, and 93.1%, respectively, was observed due to the high temperature of extraction (80 °C). While the extract obtained at 60 °C was higher for TTP, CGAs, and RSA (21.4, 37.3, and 94.4%, respectively) due to the thermal sensibility of polyphenols [41]. In bread, CGAs addition was found to improve overall key parameters. First, the addition of 2% GCE improved the bread volume (565 cc) compared to the control (525 cc) due to polyphenols’ interaction with gluten proteins and starch giving more tenacity and extensibility to the dough. GCE addition increased the greenness of bread crumb and reduced yellowness and lightness. This effect was expected because the green color indicates CGAs hydrolyzation and thermal degradation [41]. The texture of the bread was not significantly affected by GCE addition; however, the bread containing 2% GCE was softer (4.38 N) compared to the control (4.81 N). The content of TPP, RSA, and CGAs in bread significantly increased in all treatments; for TPP, the content was 0.16, 0.25, and 0.34% (for 1, 1.5, and 2% GCE, respectively) compared to the control (0.02%). RSA content increased from 12.7% for the control to 68.5% at the highest level of GCE addition. CGAs content increased from 0.28 to 0.54% with the treatments, whereas CGAs were not detected in control. Finally, for the sensory evaluation, the authors reported that despite the benefits of GCE addition in all three levels, the maximum level of enrichment without affecting the overall quality of the bread (especially taste) was 1.5% GCE.

The influence of addition of green coffee extract (GCE) prepared in aqueous extraction at 110 °C for 15 min with high content of chlorogenic acids in fried doughnuts was analyzed [175]. The GCE had a content of 25.5 g/100 g of polyphenols. The most abundant polyphenol was 5-CQA, two isomers of 5-CQA hydrolyzation (3-CQA and 4-CQA), and another ferulic compound (5-FQA). Results indicated a significant increase of antioxidant activity up to 37, 45, and 50% using three addition levels (0.25, 0.50, and 1%, respectively) compared to the control. The authors compared this enhancement with another extract from a different source of chlorogenic acids (green tea extract) GTE, and they found that GTE addition increased antioxidant activity by 22, 28, and 29% compared to the control. These data confirm that green coffee remains the vegetal species with the highest concentration of polyphenols, for example, CGAs. However, it is relevant to consider that the frying process reduces the 5-CQA concentration at the final by hydrolyzing the diesters to monoesters.

Moreover, the addition of raw coffee beans in food products has been attempted. Another study on wheat bread properties was developed by Zain et al. [185]. The authors proposed to use grounded green coffee beans (GCB) instead of an extract to substitute the flour at three levels of addition (3, 5, and 7% GCB). After baking, there was a significant increase in the TPP content since the addition of 3, 5, and 7% GCB increased TPP in bread up to eight times at the highest level (1.61 mg GAE/g) compared to control (0.26 mg GAE/g) and even at the first level (3% GCB) TPP content was nearly twice as much compared to whole wheat bread. Since polyphenols were detected in control wheat bread, the authors concluded that it was the ferulic acid present naturally in wheat flour after the milling process and amino acids and smaller peptides formed in proteolysis of protein wheat flour during fermentation of bread. Antioxidant properties of the bread were also analyzed, and they found that with GCB addition, the RSA increased significantly (up to three times more) than the control. As explained by several authors, the increase in antioxidant activity is mainly attributed to the presence of phytochemicals in GCB (mainly chlorogenic acids). However, GCB addition did affect the sensory properties of bread. According to the sensory evaluation (shape, texture, attractiveness, color, chewability, odor, and taste) for control, the overall score was around 7.3–7.5, whereas for 3, 5, and 7% GCB bread, the scores were significantly lower (6.3, 5, and 4.2, respectively). Color and volume of bread were negatively affected; the more GCB was added, the color of the crumb and crust turned green and reduced volume. This suggested that CGB addition probably affects the stability of the gluten matrix, making a compact structure. It is well known that bread types depend on cultural and geographic requirements. Therefore, enriched food formulation must be designed according to the target market [186].

The fortification of dairy products has increased in the last couple of decades [187]. A study on the field of fortification of dairy products using encapsulated green coffee extract was made by Rahpeyma et al. [179]. The extract was obtained with boiling water at 110 °C for 30 min, cooled at room temperature and filtered. The authors used an emulsion microencapsulation technique using glycerol monostearate (GMS) and canola oil, and shaking at 4000 rpm and 70 °C. They used three levels of GCE addition (0.25, 0.5, and 1%) and three levels of encapsulated green coffee extract (EGCE) at 1.25, 2.5, and 5% EGCE. The TPP content and RSA of the extract were found to be 39 and 74%, respectively. Under the microencapsulation condition, the coating material greatly impacted the retention of phenolic compounds and antioxidant activity of the extract when added to kashk. The enrichment of kashk does not affect rheological properties such as viscosity across all treatments. Noteworthily, acidification by GCE, EGCE, and lactic fermentation caused a decrease in the negative electric charge of the micelles by degrading the calcium and inorganic phosphates.

Another study on dairy products was developed by Dönmez et al. [188]. They added green coffee powder (GCP) at two levels (1 and 2%) into homemade yogurt to analyze the polyphenols´ activity and interaction with proteins from yogurt. The coffee addition reduced the serum release rate (syneresis) in yogurt. The serum separation was significantly restricted by half of the control rate with the highest addition level of GCP. Polyphenols are highly reactive to proteins since they can form protein-polyphenol complexes through multiple weak interactions (mainly hydrophobic, van der Walls, and hydrogen bridge-binding) formed between protein side chains and polyphenol aromatic rings [189]. However, interactions between GCP and proteins were strengthening the gel structure of yogurt and hence, affected its rheological behavior. The yogurt consistency was increased during the first 14 days of storage. There was no significant change in flow index of yogurt with the highest level of GCP during 21 days of storage. Color significantly changed with GCP addition; green color increased over storage time up to 40% of the greenness for day 21.

In dairy-free milk-based products, the effect of CGA addition was investigated by Seczyk et al. [177] with green coffee phenolics (CGAs) added to soymilk. The extract was obtained by aqueous heat-assisted extraction using 10 g of green coffee, boiled in 100 mL of water at 110 °C, allowed to cool down at room temperature for 1 h with continuous orbital shaking and then filtered (Whatman No. 4). Six levels of addition were used in the study (0.0025, 0.05, 0.1, 0.25, 0.5, and 1 mg of phenolics (GAE, gallic acid equivalent) per 1 mL). Compared to the control, the content of phenolics increased up to 70% at the highest level of addition of CGAs. Antioxidant activity and reducing power were significantly affected, increasing the content up to 3.5–13.8 times more than the control.

Furthermore, CGAs addition also improved the digestibility of starch and proteins in soymilk, increasing digestibility up to 17.9% higher than the unfortified soymilk. CGE addition improved soymilk aroma and texture, with grass-lemon notes. The taste was positively affected by CGAs addition showing a score range between 5–5.3 whereas the control had a 4.7 score; however, at the highest level of fortification, the taste score significantly decreased (3.0) [177].

## 7. Future Perspectives

Coffee chlorogenic acids have multifunctional properties as phytochemical and nutraceutical. Because of these properties, 5-CQA received considerable attention for its potential functional effects. With the recent advances in food fortification and the growing research interest in CGAs from different sources, its use as a natural additive to increase the intake of phenolic compounds is attractive for the food industry. There are different methods to recover CGAs; however, these extraction methods are conditioned by their physical and chemical sensitivity. Extraction methods such as electric field pulse and activated carbon purification might be used to reduce the use of toxic solvents and high temperatures, which could contaminate and reduce CGAs content in the extract. However, more research on optimization for obtaining solvent-free coffee chlorogenic acid extracts is needed.

The potential to fortify foods with CGAs would represent an option for delivering antioxidant concentrations beneficial to sustain wellbeing and health in humans, pets, and farm animals. Due to CGA’s susceptibility to temperature, pH, and light, some food processes are still challenging since they will reduce the content of CGAs in the final product. Several studies demonstrated increased antioxidant activity without affecting key quality parameters in baked products. CGAs are highly stable in yogurt and soymilk, enhancing the flavor and color while increasing their antioxidant activity. However, for products such as chocolate, more research is needed since CGAs addition significantly affects the flavor; for this reason, more research on the encapsulation of CGAs by different methods needs to be conducted to decrease the bitterness of foods enriched with CGAs is needed.

## Figures and Tables

**Table 1:** Main sources of CGAs.

Source	Concentration (g/100 g) ^1^ (dm)	CGA Composition	References
Artichoke	1–8	5-CQA, 1,5-DCQA 3,4-DCQA and DCQA	[24]
Artichoke leaves	0.92	CA, 3-CQA, 4-CQA, 5-CQA, 3,4-DQA, 3,5-DQA and 4,5-DQA	[23]
Sweet potato leaves	-	3-CQA, 3,4-DCQA, 3,5-DCQA, 4,5-DCQA and 3,4,5-TCQA	[15]
White tea (*Camelia sinensis*) leaves	1.64	3-CQA, 4-CQA, 5-CQA, 3,4-DQA, 3,5-DQA and 4,5-DQA	[23]
Green tea (*Camelia sinensis*) leaves	1.32	3-CQA, 4-CQA, 5-CQA, 3,4-DQA, 3,5-DQA and 4,5-DQA	[23]
Yerba mate (*Ilex paraguariensis*) leaves and thalli	9.19	3-CQA, 4-CQA, 5-CQA, 3,4-DQA, 3,5-DQA and 4,5-DQA	[23]
Green coffee beans	4.10–11.3 ^2^	CQA, FQA and DCQA	[25]
Apples	0.38 0–0.2 g/L (juice)	3-CQA, 5-CQA, 4,5-DCQA	[26]
Pears	0.28 0–0.24 g/L (juice)	3-CQA, 5-CQA, 3,6-DCQA	[27]
Blueberries	2	5-CQA, 3-FQA	[28]
Grapes	0.15	5-CQA, CoQA	[29]
Spinach	0.2	*p*-CoQA	[30]
Beans and peas	0.12	*p*-CoQA	[31]
Stone fruits	0.01–0.6	*p*-CoQA, 5-CQA, FQA, 4,5-DCQA, 3,4-TCQA	[32]
Potato tubers	0.5–1.2	CQA; DCQA	[33]

^1^ Units may have been changed for consistency and expressed in dry matter (dm). ^2^ It depends on the variety and geographic origin of the coffee.

**Table 2:** Principal incorporation of coffee extracts with CGAs into food products ^a^.

Food Product	Technological Improvement	Extract Conditions	CGAs Content in Green Coffee Extract	Major Findings	Sensory Evaluation	References
Fried doughnuts	Dough stability	Heated at 110 °C for 15 min and Freeze-dried	25.5 g/100 g	Dough stability was not affected during mixing and GCA showed high stability increasing antioxidant activity	No significant difference up to 1% of GCE addition (Score 5–4.9)	[175]
Instant coffee	Fortification	Heated at high pressure at 180 °C for percolation and extraction	14.0 g/100 g	Enriched coffee with green coffee extract showed high antioxidant potential but decreased sensory score	No significant difference in *C. arabica* samples (Score 7.3–6.8)	[176]
Soymilk	Fortification	Heated aqueous extraction (1:10 *w*/*v*) at 100 °C for 1 h	N.A.	Phenolic compounds and antioxidant activity content increased significantly, and overall digestibility improved	No decrease in the acceptance level up to 0.25 mg/mL of CGA (Overall score 4.3–5.2)	[177]
Wheat bread	Dough stability and fortification	Heated aqueous extraction at 60, 70 and 80 °C for 1 h	37.3 g/100 g	GCE addition increased CGAs and antioxidant activity in bread, baking quality was not affected.	Maximum level of GCE without adverse effect was 1.5% flour basis (Overall score 64–60)	[178]
Liquid Khask	Enrichment	Heated aqueous extraction (1:10 *w*/*v*) at 100 °C for 30 min and encapsulated with water and oil emulsion	39.1 g/100 g	Encapsulated GCE protected color. pH remained unaffected and rheological properties were not affected and antioxidant activity highly increased	No significant difference up to 1% of encapsulated GCE addition(Score 4.7–4.9)	[179]
Dark chocolate	Enrichment	Heated aqueous extraction (1:5 *w*/*v*) at 80 °C for 30 and encapsulated	N.A.	Addition of CGAs (free and encapsulated) had no significant effect on dark chocolate color. However, the addition of free or encapsulated CGAs had a significant effect on chocolate flavor. This adverse effect of CGAs on chocolate flavor were lower in the case of encapsulated form addition	No significant difference in the bitterness up to 50.1 mg/5 kg of encapsulated CGAs (Score 1.5–2)	[180]
Yoghurt	Enrichment	Heated aqueous extraction (1:6 *w*/*v*) at 70 °C for 1 h. The extract was filtered and concentrated by evaporation (70 °C, 30 min) and spray drying	46.5 g/100 g	Green coffee-enriched yoghurt have desirable pH (4.7), acidity, color, and minimum syneresis. The flavor, texture and other sensory attributes of yoghurt were improved.	Higher score in overall acceptance up to 2% *w*/*v* of GCE	[181]

^a^ GCE, green coffee extract; GCA, green coffee addition; N.A., not available.

## Data Availability

Not applicable.
